# Berberine Attenuates Macrophages Infiltration in Intracranial Aneurysms Potentially Through FAK/Grp78/UPR Axis

**DOI:** 10.3389/fphar.2018.00565

**Published:** 2018-05-30

**Authors:** Kai Quan, Sichen Li, Dongdong Wang, Yuan Shi, Zixiao Yang, Jianping Song, Yanlong Tian, Yingjun Liu, Zhiyuan Fan, Wei Zhu

**Affiliations:** ^1^Department of Neurosurgery, Huashan Hospital, Fudan University, Shanghai, China; ^2^Department of Radiology, Huashan Hospital, Fudan University, Shanghai, China

**Keywords:** Berberine, Focal adhesion kinase, endoplasmic reticulum stress, intracranial aneurysm, macrophage

## Abstract

**Background:** Inflammatory cells, such as macrophages, play key roles in the pathogenesis of intracranial aneurysms (IAs). Berberine (BBR), an active component of a Chinese herb *Coptis chinensis French*, has been shown to have anti-inflammatory properties through suppressing macrophage migration in various inflammation animal model. The goal of this study was to examine BBR’s effect on inflammation and IAs formation in a rodent aneurysm model.

**Methods and Results:** Human aneurysm tissues were collected by microsurgical clipping and immunostained for phospho-Focal adhesion kinase (FAK) and CD68^+^ macrophages. A rodent aneurysm model was induced in 5-week-old male Sprague Dawley (SD) rats by intracranial surgery, then these rats were orally administrated 200 mg/kg/day BBR for 35 days. Immunostaining data showed that BBR inhibited CD68^+^ macrophages accumulation in IAs tissues and suppressed FAK phosphorylation. In lipopolysaccharide (LPS)-stimulated RAW264.7 cells, BBR treatment remarkably attenuated macrophages infiltration, suppressed the expression of matrix metalloproteinases (MMPs), and reduced proinflammatory cytokine secretion, including MCP-1, interleukin 1β (IL-1β), interleukin 6 (IL-6) and tumor necrosis factor-a (TNF-α). Mechanistically, BBR downregulated FAK/Grp78/Unfolded Protein Response (UPR) signaling pathway in RAW264.7 cells.

**Conclusion:** BBR prevents IAs formation potentially through inhibiting FAK phosphorylation and inactivating UPR pathway in macrophages, which causes less macrophage infiltration and reduced proinflammatory cytokine release.

## Introduction

Intracranial aneurysm (IA), a cerebrovascular disorder in which weakness in the wall of a cerebral vessel leads to a localized dilation or ballooning of this vessel, is relatively common with a prevalence between 3–5%. Despite recent advances in diagnosis and treatment, the mortality and morbidity associated with subarachnoid hemorrhage from ruptured intracranial aneurysms still remain high (Chalouhi et al., 2013). Current standard treatments for IAs are restricted to invasive therapies, such as neurosurgical clipping and endovascular coiling treatment, both of which carry an unavoidable risk of procedural morbidity.

Several risk factors, such as hemodynamics, inflammation, and genetics, have been proposed to be linked with IAs (Nakaoka et al., 2014). Among them, mounting clues to inflammation as the dominant factor in the pathogenesis of IAs (Chalouhi et al., 2013). Because macrophages are universal finding in human intracranial aneurysm samples, they have gained significant attention in the IAs field. In addition, the infiltration of macrophages secrete proinflammatory cytokines and matrix metalloproteinases (MMPs), which recruit additional inflammatory cells and destroy extracellular matrix of arterial vessel wall to cause thinning of the blood vessels, respectively (Tada et al., 2010). Thus, targeting inflammatory macrophages may provide a promising new avenue in the treatment of IAs.

Given the critical role of inflammation in aneurysm pathogenesis, the salicylates and statins were used for preventing rupture of IAs, but there are some issues and the prognosis of rupture IAs remains miserably poor, suggesting a new therapy to prevent rupture is urgently required to address this significant unmet medical need. Berberine (BBR), a bioactive flavonoid derived from a Chinese herb *Coptis chinensis French*, has been documented to treat various indications, including arrhythmia, diabetes, hyperlipidemia, and cancer (Wang et al., 2012; Ortiz et al., 2014; Li et al., 2016). Previous reports have demonstrated the BBR’s anti-inflammatory effects are mediated by inhibiting several key signaling pathways involved in lymphocytes and macrophages infiltration, such as Nrf2 and MAPK/JNK/p38/ERK pathway (Gao et al., 2014; Mo et al., 2014), and SRC-FAK pathway (Cheng et al., 2015). Intriguingly, BBR has also been shown to have the neuroprotective activity and hypotensive effect (Zhang et al., 2012), suggesting that it could be a potential candidate stabilizing unruptured IAs.

Focal adhesion kinase, also known as PTK2, is a key cytoplasmic tyrosine kinase that plays a crucial role in integrin-mediated signaling pathway (Zhou et al., 2015). The phosphorylation of FAK causing the downstream activation of inflammatory signaling pathways including MEK/ERK1/2 (Liang et al., 2013; Liu et al., 2017). In particular, FAK has been suggested to participate in the inflammatory response induced by mechanical strain in vascular smooth muscle cells and macrophages (Harada et al., 2017).

Here, we showed that FAK was predominantly activated in macrophages within patient IAs tissues. BBR treatment markedly suppressed the development of IAs in a rat model and inhibited FAK activation. In addition, BBR reduced MMP-9 expression in macrophages and blocked macrophage recruitment by downregulating MCP-1 secretion and MCP-1- driven chemotaxis. Mechanistically, this anti-inflammatory effect was dependent on the regulation of UPR signaling pathways, which initiated the transcription of MMP-9/MCP-1. In conclusion, we propose that BBR is a potential novel therapeutic agent in IAs by controlling proinflammatory macrophages.

## Materials and Methods

### Rat Intracranial Aneurysm Model

Experiments were conducted in accordance with the guidelines and were approved by the Huashan hospital, Fudan University, Institutional Animal Care and Use Committee. Under anesthesia with chloral hydrate (400 mg/kg), adult male Sprague Dawley (SD) rats (5 weeks old) including Sham (12 rats), Vehicle (12 rats) and BBR-treated (12 rats, 200 mg/kg/day) groups. Vehicle and BBR-treated rats were subjected to left internal carotid artery ligation and performed a single stereotaxic injection (68001; RWD, Shenzhen, China) of elastase (Sigma-Aldrich, St. Louis, MO, United States) into the cerebrospinal fluid at the right basal cistern as described previously (Nuki et al., 2009). Hypertension was induced by a continuous high salt diet for 5 weeks. We using the tail cuff method to examine systolic arterial blood pressure in rats before treatment, 1 and 2 weeks after surgery. After 35 days, rats were euthanized and perfused with bromophenol blue dye in arteries, and the circle of Willis (COW) and major branches were blindly determined by two observers. The Aneurysm grade evaluate were used previous described (Hosaka et al., 2014).

### Immunofluorescence Staining

We collected human IAs specimens from patients undergoing surgical IAs Clipping, and control normal superficial temporal artery (STA) tissues. Slides were incubated with 2% bovine serum albumin before incubated with primary antibodies at 4°C overnight. After washing thrice by PBS (5 min/each time), slides were incubated with Alexa Fluor 488 or 549 conjugated secondary antibodies at room temperature for 2 h. Coverslips were mounted by a medium containing DAPI, and examined under a fluorescence confocal microscope (FV1000; Olympus, Tokyo, Japan). The primary antibodies used in immunohistochemistry were anti-CD68 (Abcam, Ab955, London, United Kingdom), and anti-phosphor-FAK (Cell signaling, 3283, Danvers, MA, United States). Staining density was analyzed by Image J software.

### Immunohistochemistry Staining

Intracranial aneurysms and STA tissues were fixed in formaldehyde then paraffin embedded and 0.5 μm sections were prepared. Immunohistochemistry were performed as previously described (Tada et al., 2010). Staining density was analyzed by Image J software.

### Cell Preparation, Culture and Treatment

RAW264.7, a mouse macrophage cell line, was obtained from Chinese Academy of Sciences (Shanghai, China) and cultured in a Dulbecco modified Eagle medium (DMEM) (Gibco, Grand Island, NY, United States) in 10% fetal bovine serum (FBS) (Gibco) and a penicillin-streptomycin solution (100 U/mL penicillin, 100 ng/mL streptomycin) (Hyclone, Logan, UT, United States) in a 37°C humidified incubator containing 5% CO2. LPS-induced RAW264.7 cells were seeded in a 24-well plate at a density of 1 × 10^5^ cells per well, and the cells were stimulated with or without 10 μM lipopolysaccharide. If it is needed, cells were pretreated with 10 μM PF573228 (a FAK inhibitor, Selleck, Houston, TX, United States) for 1 h and berberine (20 μM, Sigma-Aldrich, St. Louis, MO, United States) for 2 h, followed by treatment with Tunicamycin (TM, 5 μg/ml, Sigma-Aldrich, St. Louis, MO, United States) or 4-Phenylbutyric acid (4-PBA, 1 mM, Sigma-Aldrich) for 10 min.

### Migration Assay

Cell migration was measured by determining the number of cells that migrated through 8 μm pore transwell. Briefly, after 24 h incubation, cells migrated into the lower wells through the pores were quantitated by gentian violet assay, and presented as the total cell numbers in the lower wells.

### Adhesion Assay

Plates were coated with 40 μg/ml Collagen I at 4°C for 12 h, and 100 μl RAW264.7 cell suspension was loaded into each well. Non-adherent cells were washed by 100 μl DMEM, and the plate was read on a spectrophotometer to measure absorbance at 570 nm.

### Western Blotting

For Western blotting experiments, RAW264.7 cell lysates were separated by SDS–PAGE and then detected by the following antibodies: anti-GAPDH (Kanchen Bio-tech Corporation, KC-5G5, Shanghai, China), anti-FAK (Abcam, Ab40794, London, United Kingdom), anti-phospho-FAK (Abcam, Ab81298), anti-MMP2/9 (Abcam, Ab92536/ Ab76003), anti-Grp78 (3177), anti-PERK (5683), anti-phospho-PERK (3179), anti-Xbp-1s (12782), anti-p50-ATF6 (65850), anti-Histon H3 (9715). Other antibodies were purchased from Cell Signaling (Danvers, MA, United States). The membrane was incubated with enhanced chemiluminescent detection kit (Amersham, London, United Kingdom), exposed to X-ray film, and quantified by a Gel Doc 2000 system (Bio-Rad, Hercules, CA, United States).

### Detection of IL-1β, IL-6, MCP-1, and TNF-α

BBR, PF573228, TM, or PBA was added to RAW264.7 cells before the LPS stimulation. Supernatant cytokine and chemokine levels (IL-1β, IL-6, MCP1, and TNF-α) were measured by enzyme-linked immunosorbent assay (ELISA) kits from R&D Systems (Minneapolis, MN, United States) according to the manufacturer’s protocols.

### Ethics Approval

The use of human IA specimens and control arterial walls was approved by the ethical committees of Huashan hospital, Fudan University (IRB2017-398).

### Statistical Analysis

Data were presented as mean ± SEM and analyzed among groups using the Student’s *t*-test or the Wilcoxon rank sum test. *P* < 0.05 was considered statistically significant.

## Result

### Robust FAK Phosphorylation and More Infiltrated Macrophage in Patient’s IAs Tissue

**FIGURE 1:**
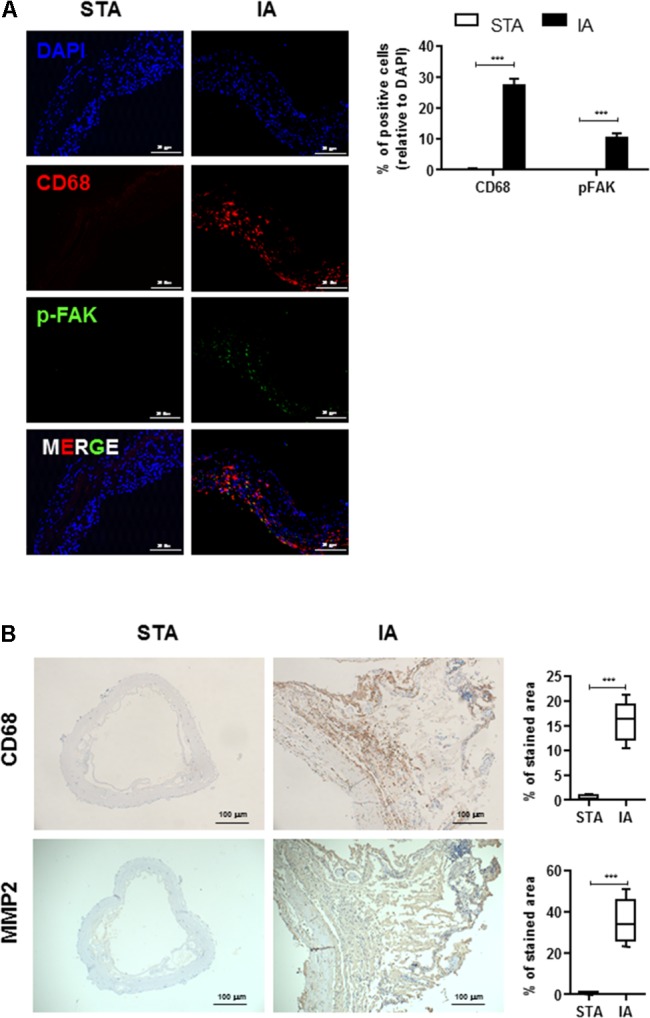
Infiltrated CD68^+^ macrophages are co-localized with phospho-FAK signal in human intracranial aneurysms (IAs) tissues. **(A)** STA and IAs were double-stained using DAPI (blue), anti-CD68 (red) and anti-pFAK (green) and found CD68^+^ macrophage cells were largely colocalized with phospho-FAK. 20 μm scale bar was shown. **(B)** Compared with superficial temporal artery (STA), CD68^+^ macrophage cells and MMP2 were abundant in adventitia of IAs. 100 μm scale bar was shown. ^∗∗∗^*P* < 0.001.

To determine whether FAK was activated in IAs tissues, we first determined the levels of phosphorylated FAK and CD68 in human IAs specimens from patients undergoing surgical IAs Clipping, and control normal superficial temporal artery (STA) tissues. Although no signal was detected in STA, phospho-FAK, an activated form of FAK, was mainly detected in the medial and adventitial layers of IAs walls and was frequently observed near severe inflammatory cells infiltration and tissue destruction (**Figure 1A**). In particular, activated FAK largely colocalized with CD68-positive macrophages in the adventitia (**Figure 1A**). We also determined the localization of MMP2 and its association with pathological tissue changes in human IAS walls. CD68 and MMP2 was significantly increased in vascular adventitia in human IAs’ tissues compared with control STA tissues (**Figure 1B**), indicating macrophages mainly infiltrated into adventitia of IAs’ walls.

### Administration of BBR Prevented Aneurysm Formation and Reduced Tissue-Infiltration Macrophages in Rat Experimental IAs

**FIGURE 2:**
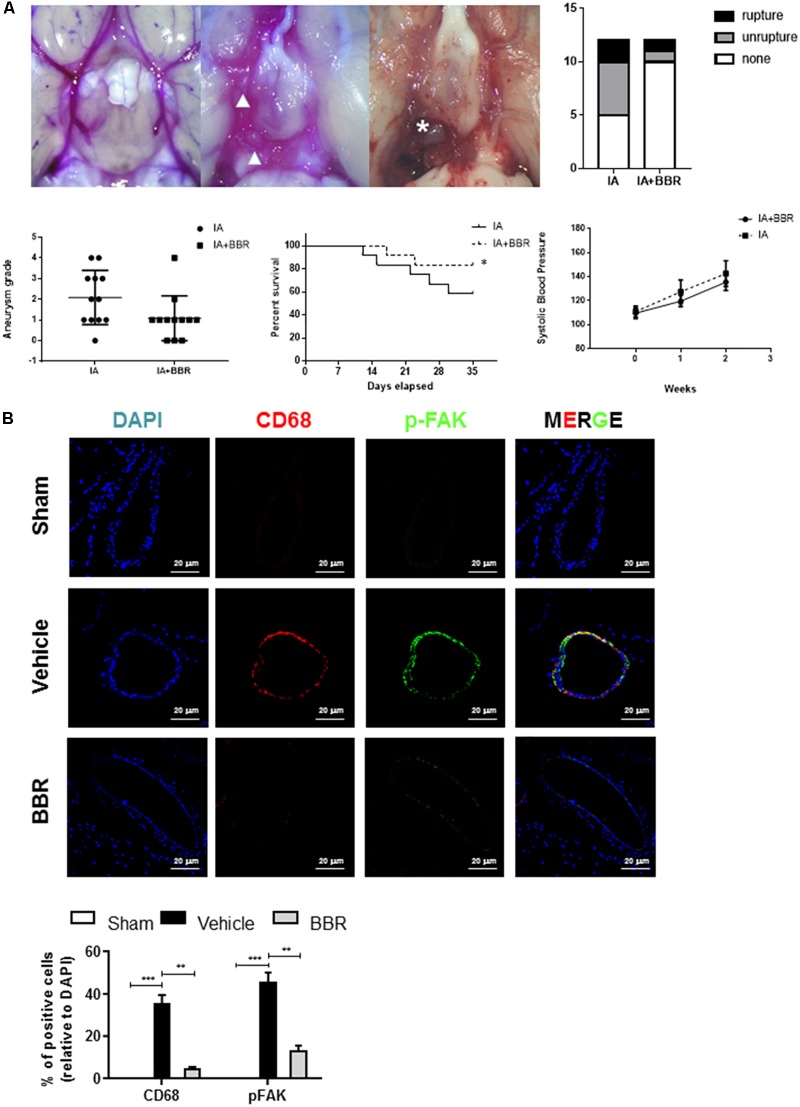
BBR treatment suppresses IAs formation and macrophage infiltration in SD-rat model. **(A)** BBR treated animals show less IA formation (arrowhead) and less vessel rupture (asterisk). Representative images of cerebral arteries at 35 days and quantification of IAs formation (triangle) and rupture (asterisk). Grade 0: normal arteries; Grade 1: abnormal/aneurysmal arteries; Grade 2: one aneurysm within the COW; Grade 3: multiple aneurysms within the COW; Grade 4: ruptured aneurysm. **(B)** IAs were double-stained using DAPI (blue), anti-CD68 (red) and anti-pFAK (green). 20 μm scale bar was shown.

To further examine FAK activation in an experimental model of IAs, we used a SD rat model of IA involving stereotaxic injection of elastase into the cerebrospinal fluid. This surgery induced inflammatory cell infiltration and the gradual destruction of elastic lamellae in the cerebral artery wall (Tada et al., 2010; Hoh et al., 2014). As shown in **Figure 2A**, BBR treatment group demonstrated less formation of ruptured and unruptured IAs, and there was no significant difference in the systolic blood pressure among the groups at each time point. Monocyte-macrophages infiltration and activation play crucial roles in chronic inflammation and are related to the development of IAs (Nuki et al., 2009; Chalouhi et al., 2012). Consistent with our findings in the walls of human IA (**Figure 1A**), phospho-FAK and CD68 signals were increased in the cerebral aneurysms walls of Vehicle group compared with those of sham group (**Figure 2B**). CD68^+^ macrophages had less infiltration into the IAs of the BBR-treated group than those in the vehicle and sham group. Accordingly, phospho-FAK expression in cerebral arteries was significantly decreased in rats treated with BBR (**Figure 2B**). Therefore, we noticed macrophages as the main target cells of BBR in the following study.

### Phosphorylation of FAK Is Involved in BBR Suppressed Macrophage Migration and Inflammatory Response in RAW264.7 Murine Macrophage-Like Cells

**FIGURE 3:**
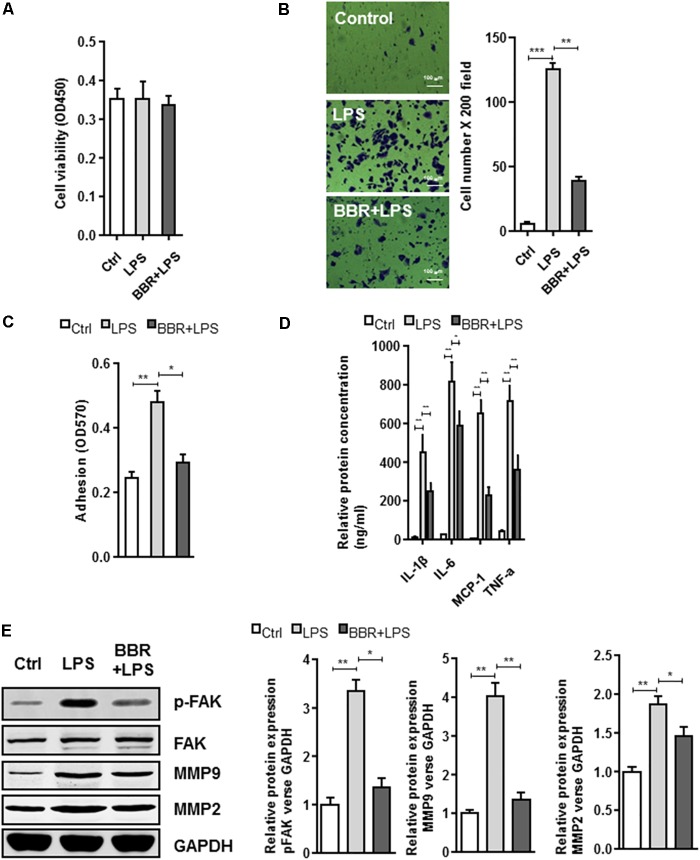
*In vitro* effect of BBR on cell migration and inflammatory response in LPS-induced RAW264.7 cell. **(A)** RAW264.7 cell viability assay was conducted after cells were treated with BBR. **(B)** Transwell assay was performed to determine the invasiveness of the cells after treatment. **(C)** adhesion assays was examined after indicated treatment. **(D)** Inflammatory response of lipopolysaccharide (LPS)-stimulated RAW264.7 cells was analyzed using ELISA assay after indicated treatment. IL-1β, IL-6, MCP-1, and TNF-α are significantly downregulated in the BBR treated group. **(E)** After LPS pretreated RAW264.7 cells were incubated with BBR for 24 h, the protein levels of FAK, p-FAK, MMP2, and MMP9 (left) were determined by Western Blot assay. The corresponding quantified data were shown in the right panel. Results were presented as mean ± SEM. ^∗^*P* < 0.05, ^∗∗^*P* < 0.01, ^∗∗∗^*P* < 0.001 vs. control group.

**FIGURE 4:**
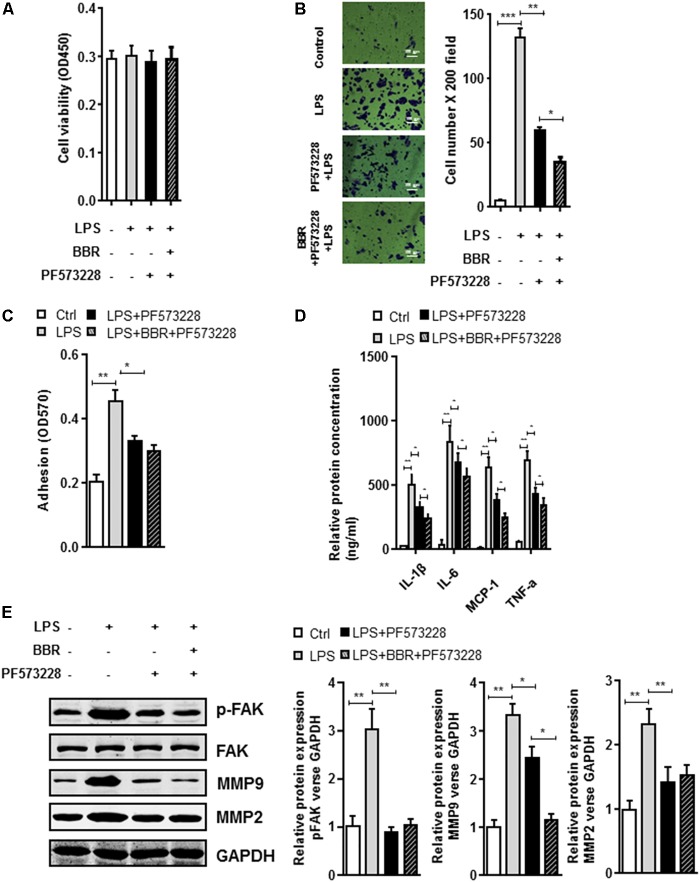
*In vit*ro synergistic effect of PF573228 and BBR on cell migration and inflammatory response in LPS-pretreated RAW264.7 cells. **(A)** Cell viability assay was performed after RAW264.7 cells were treated with BBR and PF573228. **(B)** Transwell assay was performed to determine the invasiveness of the cells after BBR treatment and PF573228 treatment. **(C)** Adhesion assay was examined after indicated treatment. **(D)** Inflammatory response of LPS-stimulated RAW264.7 cell was analyzed using ELISA assay after indicated treatment. IL-1β, IL-6, MCP-1 and TNF-α levels were significantly downregulated in the PF573228 treated group. **(E)** After LPS-pretreated RAW264.7 cells were incubated with PF573228 for 24 h, the protein levels of FAK, p-FAK, MMP2, and MMP9 (left) were determined by Western blot assay. The corresponding quantified data were shown in the right panel. Results were presented as mean ± SEM. ^∗^*P* < 0.05, ^∗∗^*P* < 0.01, ^∗∗∗^*P* < 0.001 vs. control group.

The BBR’s anti-inflammatory effect was detected using LPS-stimulated RAW264.7 cells (**Supplementary Figure S1**). Transwell assay was conducted to examine cell migration. Compared with the control group, BBR had no effect on cell proliferation, but effectively reduced RAW264.7 cells to migrate to the bottom well (**Figures 3A–C**). Moreover, BBR’s treatment attenuated the increasing of proinflammatory cytokines caused by lipopolysaccharide, including IL-6, TNF-α, IL-1β, and MCP-1 (**Figure 3D**). BBR-treated cells had impaired FAK phosphorylation and less MMP 2/9 expression (**Figure 3E** and **Supplementary Figure S2**), indicating that BBR blocked the inflammatory activation of macrophages via the FAK pathways. The elevated FAK activity observed s in IAs (**Figure 1A**) and BBR’s effect on pFAK prompted us to examine the link between BBR’s anti-inflammatory activity and FAK phosphorylation by taking advantage of the competitive phospho-FAK inhibitors PF573228, which was shown to regulate FAK active in tyrosine kinase-dependent manner (Wang et al., 2015). As shown in **Figures 4A–E** and **Supplementary Figure S3**, pretreatment with PF573228 exerted a same inhibitory effect as BBR on cell migration, adhesion, and pro-inflammatory cytokines production in LPS-stimulated RAW264.7 cells. Overall, these results indicated that the anti-inflammatory effect of BBR in macrophages was, in part at least, dependent on FAK phosphorylation.

### Unfolded Protein Response (UPR) Signaling Inhibition Mediates BBR Anti-inflammatory Effect in Macrophages

**FIGURE 5:**
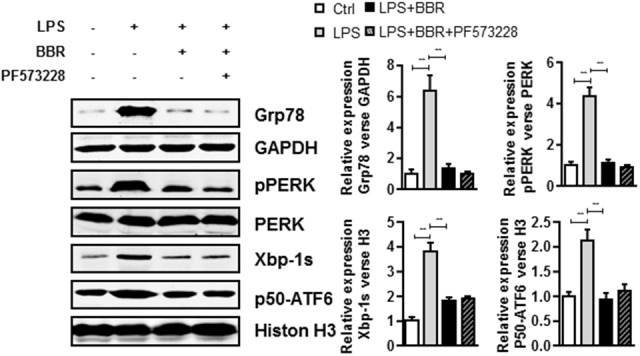
BBR and PF573228 treatment inhibits URP related proteins expression in RAW264.7 macrophages. A, LPS-stimulated RAW264.7 cells were first treated with BBR and PF573228, and western blot assay was performed to determine the URP related protein levels of Grp78, pPERK, Xbp-1s, and p50-ATF6 (left). The corresponding quantified data were shown in the right panel. Results were presented as mean ± SEM. ^∗^*P* < 0.05, ^∗∗^*P* < 0.01, ^∗∗∗^*P* < 0.001 vs. control group.

We further investigated phospho-FAK’s downstream effectors which played roles in the anti-inflammatory effect of BBR. Because 78 kDa glucose-regulated protein (Grp78) is a crucial gene for inflammatory cytokines and macrophages infiltration (Bin et al., 2017), we first examined Grp78 expression in LPS-induced RAW264.7 cells and found that LPS highly induced Grp78 expression. Importantly, BBR or PF573228 treatment impaired LPS-induced Grp78 protein (**Figure 5** and **Supplementary Figure S4**). In addition, previous studies have suggested that endoplasmic reticulum (ER) stress played an important role in inflammation response in macrophage and leukocyte (Zhou and Tabas, 2013; Du et al., 2015). The unfolded protein response (UPR), also known as the ER stress response, is a variety of highly conserved signaling pathways focused on maintain homeostasis under ER stress, including PKR-like ER kinase (PREK), activating transcription factor 6 (ATF6), and inositol-requiring enzyme 1α (IRE1α) pathways (Ozcan, 2006). In our study, all these UPR pathways were in activated in LPS-stimulated RAW 264.7 cells, as evidenced by the significant upregulation of pPERK, Xbp-1s and p50-ATF6. Similar to Grp78, BBR or PF573228 treatment remarkably abolished LPS induced UPR pathway activation (**Figure 5**).

**FIGURE 6:**
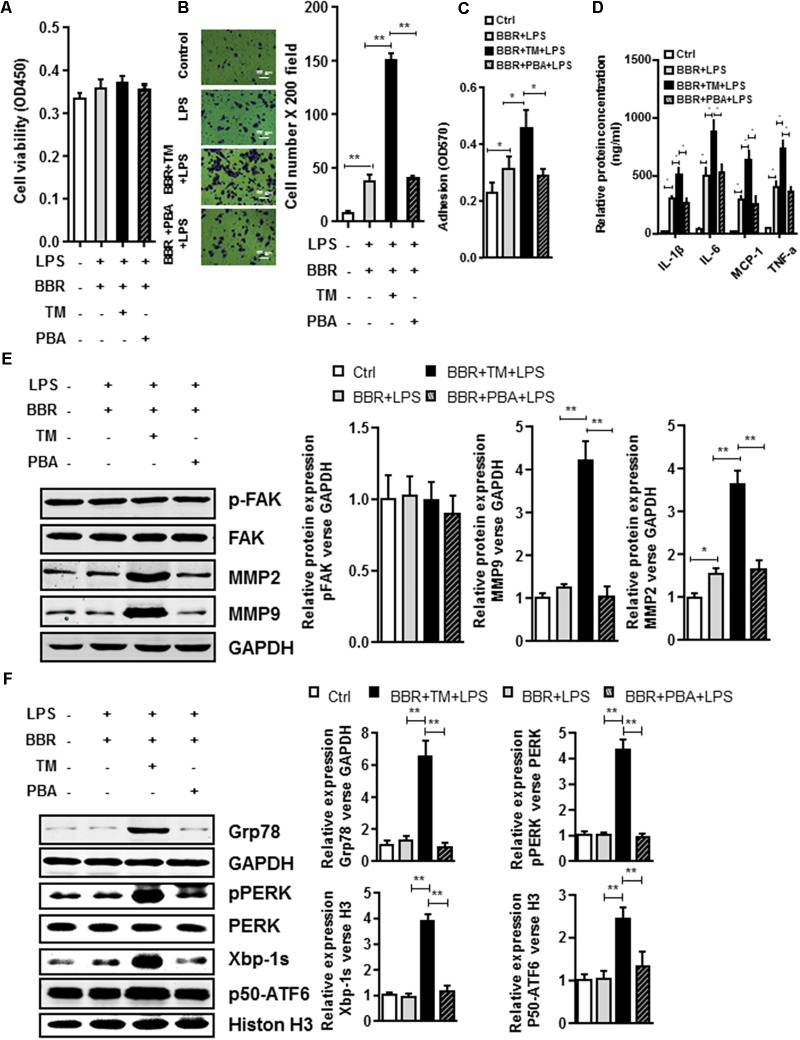
UPR signaling pathway is involved in BBR anti-Inflammatory Effect in Macrophages. **(A)** Cell viability assay was performed after RAW264.7 cells were stimulated with Tunicamycin (TM) or 4-Phenylbutyric acid (PBA) and treatment with BBR. **(B)** After RAW264.7 cells were stimulated with BBR, BBR+TM or BBR+PBA, transwell assay was performed. **(C)** Adhesion assays was examined after indicated treatment. **(D)** Inflammatory response of LPS-pretreated RAW264.7 cell was analyzed using ELISA assay after indicated treatment. **(E)** LPS-pretreated RAW264.7 cells were stimulated with TM or PBA and treatment with BBR, western blot assay was performed to measure the protein levels of FAK, p-FAK, MMP2, and MMP9 (left). The corresponding quantified data were shown in the right panel. **(F)** LPS-pretreated RAW264.7 cells were treated stimulated with BBR, BBR+TM or BBR+PBA, western blot assay was performed to measure the URP related protein levels of Grp78, pPERK, Xbp-1s, and p50-ATF6 (left). The corresponding quantified data were shown in the right panel. Results were presented as mean ± SEM. ^∗^*P* < 0.05, ^∗∗^*P* < 0.01, ^∗∗∗^*P* < 0.001 vs. control group.

**FIGURE 7:**
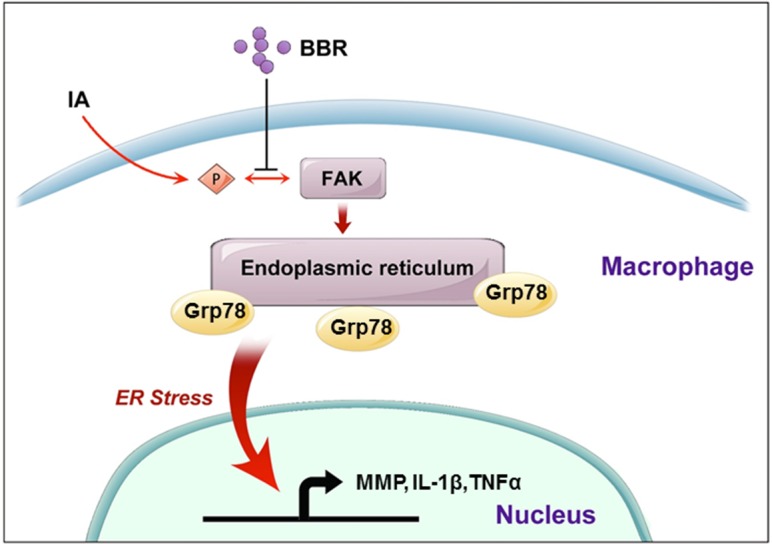
A schematic model to describe the mechanism of BBR’s anti-inflammatory effect in IAs. BBR inhibits the phosphorylation and activation of FAK, induces the downregulate of URP related proteins (Grp78, pPERK, Xbp-1s, and p50-ATF6), and suppresses the secretion of pro-inflammatory cytokines, which eventually attenuates macrophage cells active and infiltration in human IAs.

To further test the involvement of UPR response in BBR-mediated anti-inflammation process, we tried another approach by taking advantage of ER stress inhibitor (4-PBA) or inducer (tunicamycin (TM)). Although application of ER stress inhibitor 4-PBA did not affect BBR’s activity in LPS-induced macrophages (**Figures 6A,E** and **Supplementary Figure S5**), TM completely reversed BBR’s effect on UPR response in RAW264.7 macrophage cells (**Figure 6F** and **Supplementary Figure S5**), and subsequently caused significant increases in both the macrophage migration and adhesion (**Figures 6B,C**), proinflammatory cytokine production (**Figure 6D**), and the production of MMP-2/9 after 24 h (**Figure 6D**). Taken together, these results showed that BBR negatively regulated macrophage infiltration and the secretion of MMP-2/9 by suppressing ER stress (**Figure 7**).

## Discussion

The present study demonstrated that oral administration of BBR, inhibited macrophage infiltration, which led to the suppression of IAs growth in rodent model. Furthermore, we found that BBR’s treatment attenuated the inflammatory activation of macrophages, at least partially, through a FAK-dependent pathway. In line with previous reports, our results support the model in which BBR inhibits FAK phosphorylation in macrophages through negatively regulating Grp78 and subsequent ER stress (Tang et al., 2012).

Increasing evidence reveals that blood flow on the endothelial of the cerebral arteries causes the formation and development of IAs, triggering chronic inflammation in the vessel wall (Chalouhi et al., 2013). Repetitive inflammation involves the presence of macrophages and the subsequent production of proinflammatory factors and matrix metalloproteinases, that induce degradation of cellular constituents of the artery wall, causing the thinning of the arteries wall (Aoki et al., 2007; Chalouhi et al., 2012). Given the critical role of inflammation in aneurysm pathogenesis, several therapeutic strategies have been investigated with overall mixed but promising results (Hasan et al., 2011, 2013; Tada et al., 2011; Marbacher et al., 2012).

Focal adhesion kinase is activated by various stimuli involved in the process of vascular development, including angiopoietin-1,cyclic mechanical stretching or fluid shear stress, suggest that FAK may take a key part in the development of a functional circulation and its responsiveness to micro environmental clues (Ilic et al., 2003; Braren et al., 2006; Nimgulkar et al., 2015). To determine whether phospho-FAK or its downstream proteins were BBR’s target in macrophages, we provided some pieces of evidence from different angles. In our study, BBR-treatment not only suppressed the phosphorylation of FAK and reduced expression of phosphor-FAK targeted cytokines, but also reduced the glucose-regulated protein Grp78 in endoplasmic reticulum. In previous studies, upregulated expression of the Grp78 is the mostly used readout for UPR signaling activation, which process to facilitate cell infiltration and results in inflammation (Yang et al., 2016). Furthermore, BBR’s effect on attenuating inflammation was abolished in the presence of the ER stress activator, TM (Ozcan, 2004). Meanwhile, BBR showed a similar effects to that of the ER stress inhibitor, PBA, in suppressing inflammatory responses in macrophages (Ozcan, 2006; Zhang et al., 2016). Overall, these observations indicated that suppression of the FAK/Grp78/ UPR signaling pathway may be the mechanism to explain BBR induced anti-inflammatory effect.

The ER is an organelle responsible for secretory and membrane proteins-folding, lipid biosynthesis and calcium reserve (Ozcan, 2004). A number of conditions caused the accumulation of misfolded proteins in the ER, a phenomena referred to as “ER stress” (Fritz et al., 2011). The UPR pathways triggered ER stress, and mediated by several transmembrane proteins, including PREK, IRE1α and ATF6 (Hetz and Glimcher, 2009; Zhou and Tabas, 2013). Regarding its essential roles in vessel walls, URP activation promotes inflammation in vascular endothelial cells and plays an important role in pathological vascular remodeling (Zhou and Tabas, 2013). UPR activation in macrophages is an adaptive survival mechanism that enhances the immune damage (Bin et al., 2017). Specifically, similar to FAK inhibitor or UPR inhibitor, BBR decreased the expression of pro-inflammatory cytokines and reduced macrophages infiltration *in vitro*, indicating that BBR’s anti-inflammatory effect of BBR in the growth of IAs was mediated by phosphor-FAK and UPR.

Nevertheless, this study had several limitations. First, because BBR is an anti-inflammation drug with multiple targets, phosphor-FAK and UPR might only be a partial mechanism involved in IAs growth inhibition. Other pathways, like JAK2/STAT3 and PI3K pathway, could also play roles. Secondly, although a number of previous reports have suggested that Grp78 protein is a molecular target of phosphor-FAK, we still do not know whether Grp78 protein is a direct target of FAK. Further studies need to be performed concerning this part. Thirdly, to dissect the role of FAK or ER stress in BBR-mediated anti-inflammation process, we conducted *in vitro* experiments in RAW264.7 cells, which is murine macrophage-like cells. Further *in vivo* studies will provide more evidence to demonstrate these signaling in IA growth process.

As illustrated schematically in **Figure 7**, we demonstrated that BBR prevents the development of IAs via its anti-inflammatory effects in macrophages. Dephosphorylation of FAK and subsequent inhibition of the ER stress in macrophages may be key steps in BBR mediated anti-inflammatory mechanisms. Further investigation in pre-clinical and clinical settings may lead to develop BBR as a novel therapy for IAs.

## Author Contributions

WZ mainly conceived the project and designed the study. KQ, SL, and DW performed all experiments, participated in the experimental design, and drafted the manuscript. YS and ZY analyzed and interpreted part of the data. YT, JS, YL, and ZF mainly contributed to the conception and revised the manuscript. All authors read and approved the final manuscript.

## Conflict of Interest Statement

The authors declare that the research was conducted in the absence of any commercial or financial relationships that could be construed as a potential conflict of interest.

## Supplementary Material

FIGURE S1 Raw 264.7 cell’s LPS dose response in cell viability assay **(A)** and transwell assay **(B)**. ^∗^*P* < 0.05, ^∗∗^*P* < 0.01, ^∗∗∗^*P* < 0.001 vs. control group.

FIGURE S2 Full WB gels of **Figure 3**.

FIGURE S3 Full WB gels of **Figure 4**.

FIGURE S4 Full WB gels of **Figure 5**.

FIGURE S5 Full WB gels of **Figure 6E** **(A)** and **Figure 6F** **(B)**.
