# Supplementary figures and images for: Berberine Attenuates Macrophages Infiltration in Intracranial Aneurysms Potentially Through FAK/Grp78/UPR Axis
Source: Front Pharmacol. 2018 May 30;9:565. doi: 10.3389/fphar.2018.00565 (PMC5988844; doi:10.3389/fphar.2018.00565)

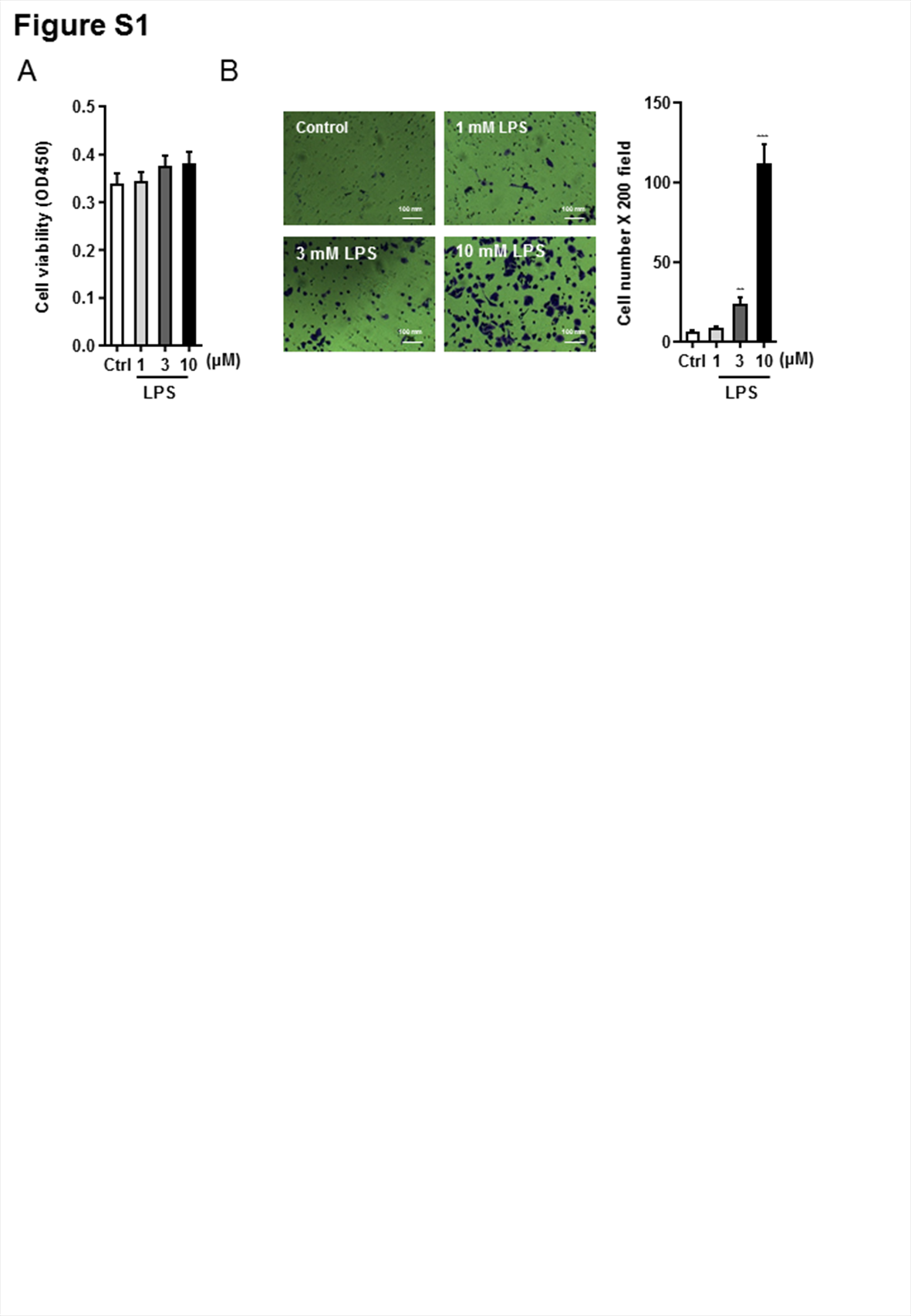

Supplement: FIGURE S1 — Raw 264.7 cell’s LPS dose response in cell viability assay (A) and transwell assay (B). ∗P < 0.05, ∗∗P < 0.01, ∗∗∗P < 0.001 vs. control group. [file Image_1.TIF]

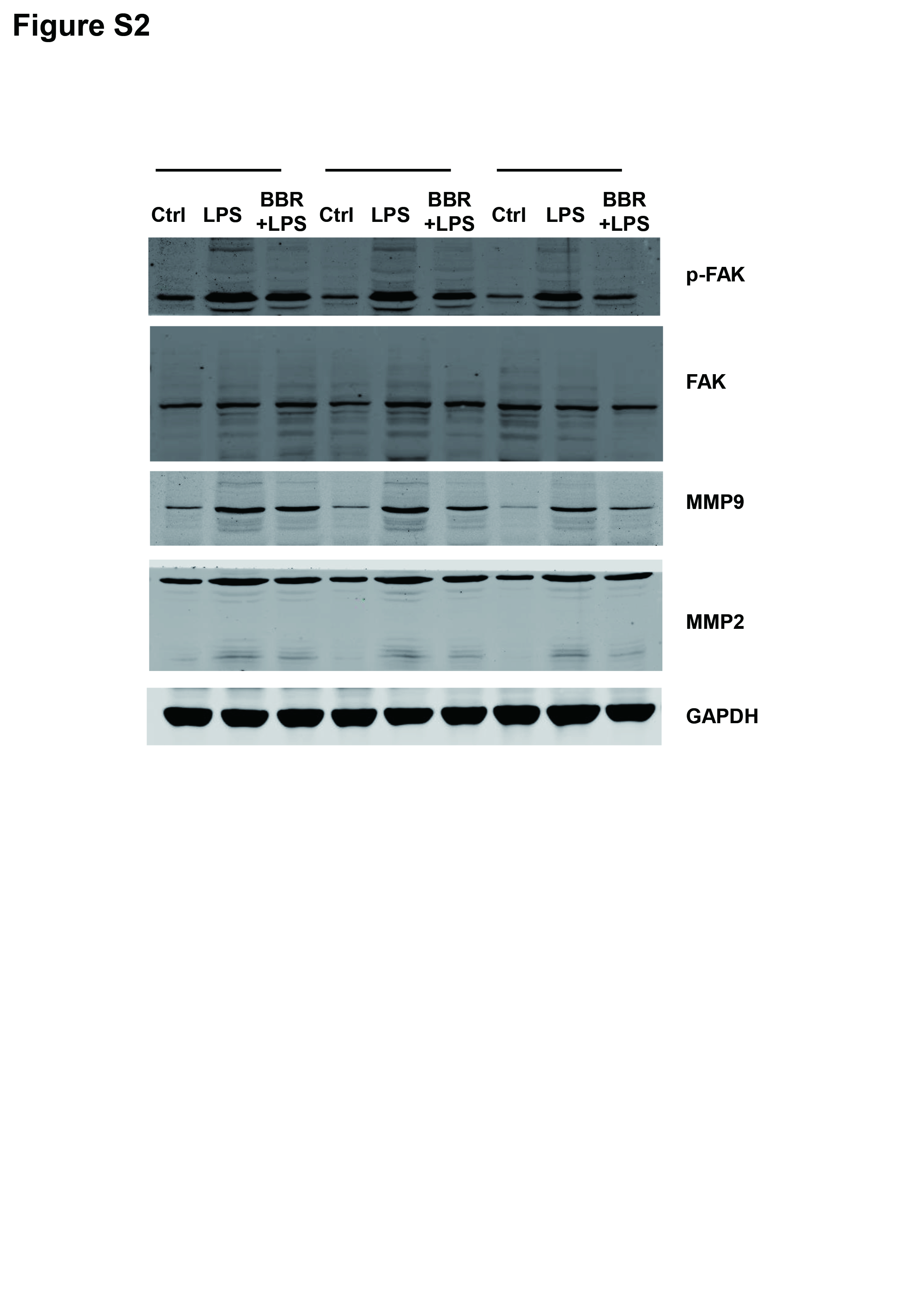

Supplement: FIGURE S2 — Full WB gels of Figure 3. [file Image_2.TIF]

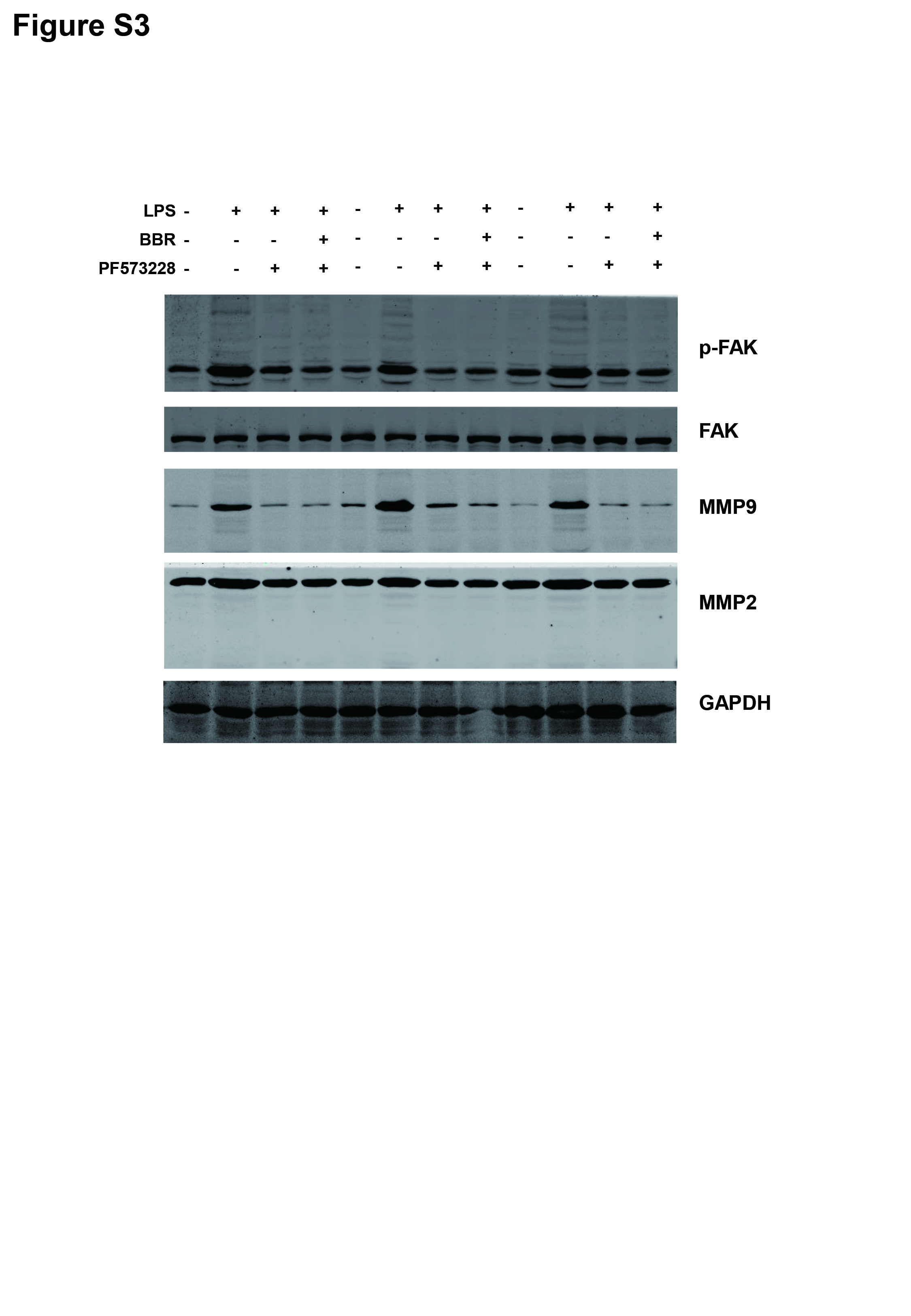

Supplement: FIGURE S3 — Full WB gels of Figure 4. [file Image_3.TIF]

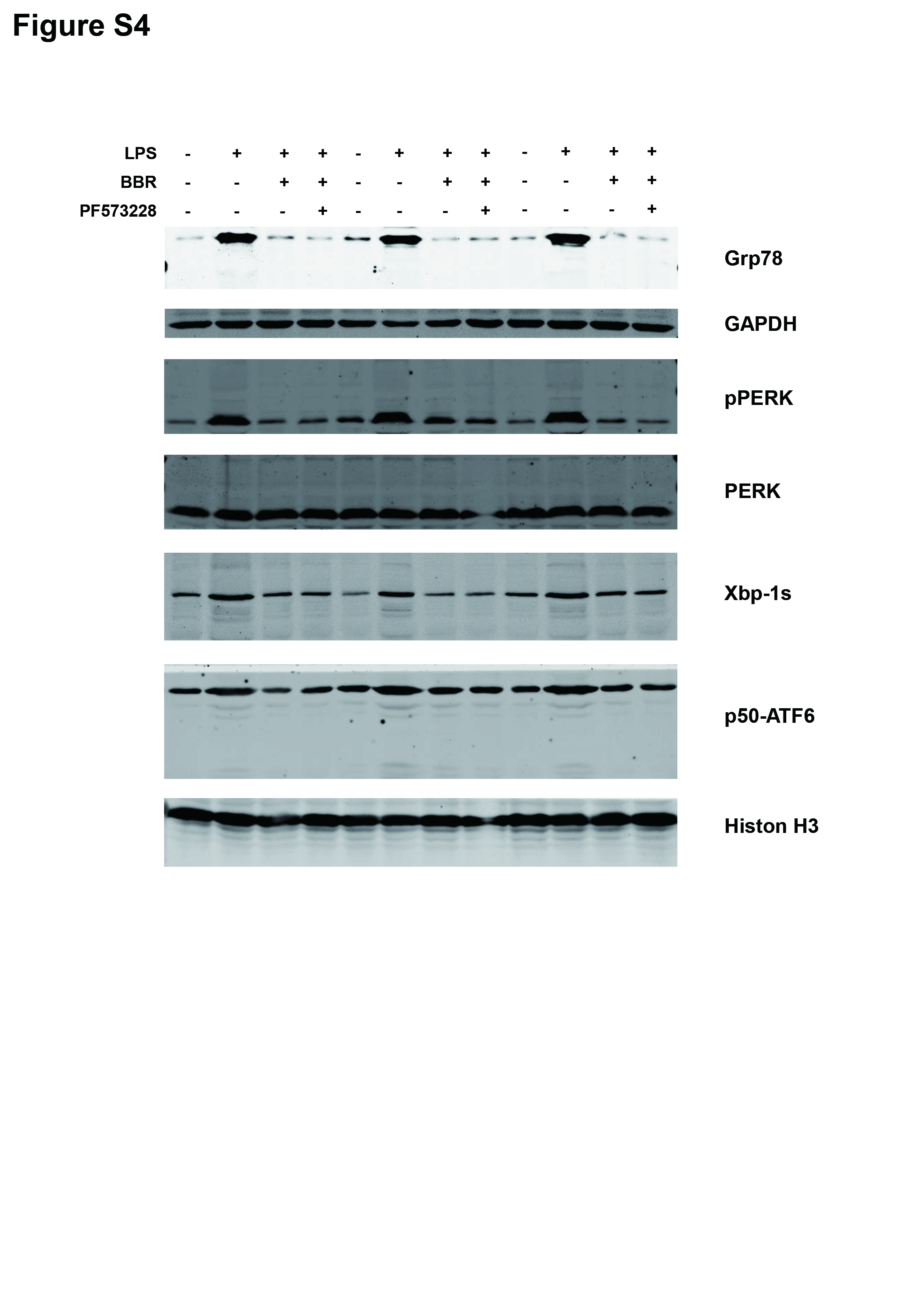

Supplement: FIGURE S4 — Full WB gels of Figure 5. [file Image_4.TIF]

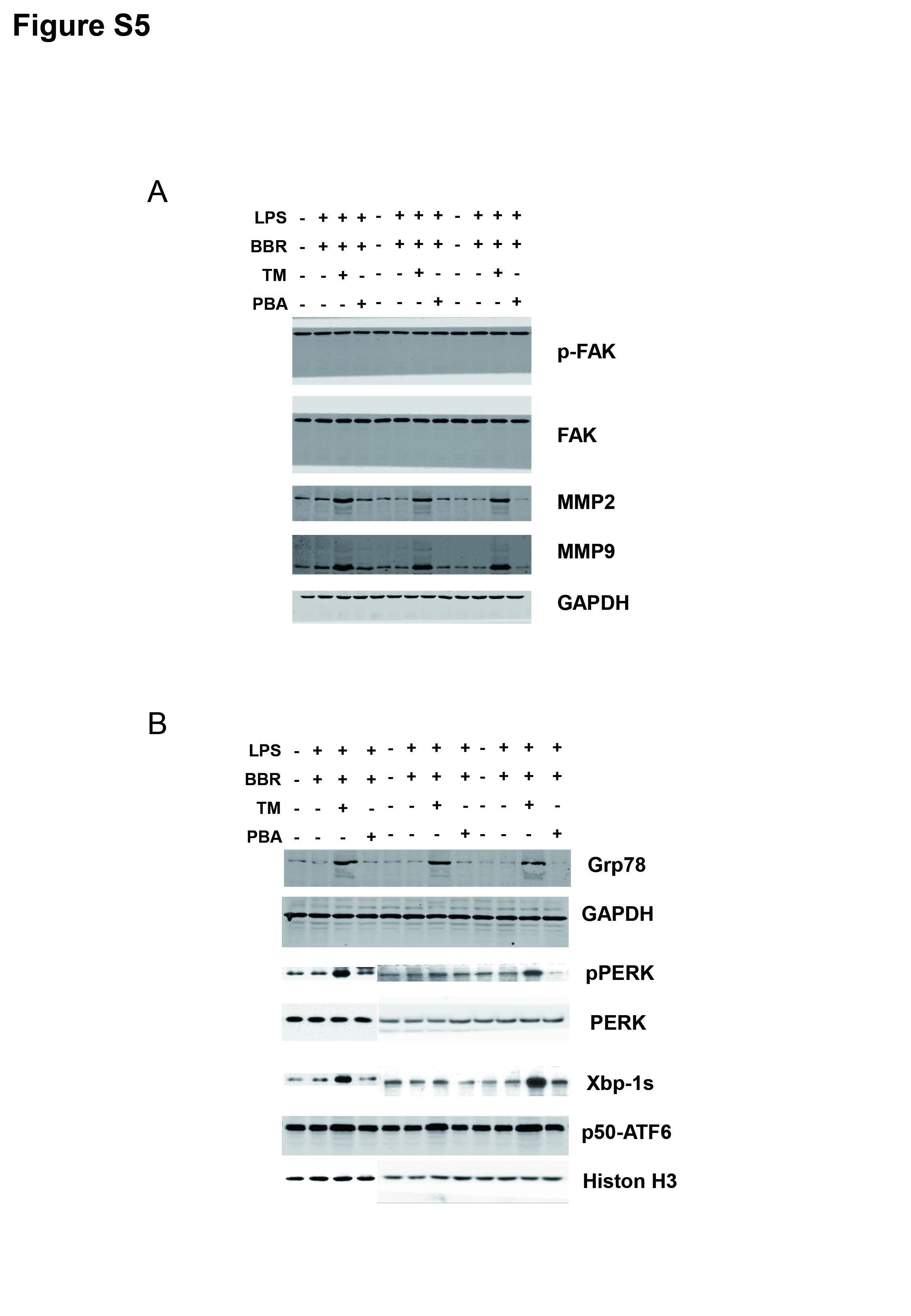

Supplement: FIGURE S5 — Full WB gels of Figure 6E (A) and Figure 6F (B). [file Image_5.TIF]
